# Mental health, Reproductive health and contraception: pharmacological interaction, current evidence and challenges

**DOI:** 10.1192/j.eurpsy.2023.2392

**Published:** 2023-07-19

**Authors:** C. Pinto Osório, P. Macedo

**Affiliations:** 1 Departamento de Psiquiatria e Saúde Mental, Centro Hospitalar do Tâmega e Sousa, Penafiel, Portugal

## Abstract

**Introduction:**

Mental pathology is relatively prevalent in the women, and there is often a need for treatment with psychotropic drugs, with an impact on women’s reproductive health. The use of contraception is widely spread among this population and the choice between these methods must be considered carefully.

**Objectives:**

This review aims to present the main interactions between contraceptive methods and psychotropic therapy and its impact on psychiatric disorders, in addition to the precautions to be taken in their choice.

**Methods:**

Literature review of relevant articles on Pubmed. Query: Contraceptive+(psychiatric disorder, depression, anxiety, schizophrenia, bipolar disorder, psychotropic drugs).

**Results:**

Based on current evidence, no statistically significant differences regarding unplanned pregnancy rates or the psychotropic treatment outcomes were found when using combined oral contraceptives and antidepressants, benzodiazepines or atypical antipsychotics (being clozapine the exception). The impact of contraceptive methods on mood was unclear, as some articles showed an association between contraceptive use and a higher risk of beginning antidepressants and others showing no differences or even as a protective factor. Although the interactions of the aforementioned drugs are infrequent, there are cases where important interactions occur, such as clozapine, valproic acid, lamotrigine and carbamazepine, as some herbal products as the St.John’s wort. With clozapine, there is an increased serum concentrations, while the opposite occurs in the case of valproate and lamotrigine with a decrease of 32.6% and 23.4%, respectively. Treatment with valproic acid in women of childbearing age has been discouraged because of its association with polycystic ovary syndrome, elevated testosterone concentrations and menstrual irregularities, in addition to the risk of fetal malformations. In cases where this drug is prescribed, it is recommended to use highly effective methods such as subcutaneous implants or intrauterine devices. Lamotrigine and carbamazepine reduce the effectiveness of some contraceptive methods, such as oral contraceptives, transdermal patch and vaginal ring, in which case the placement of a subcutaneous implant or intrauterine device is indicated.

**Conclusions:**

The magnitude of the impact between contraceptives, regarding depressive disorders, is unclear. The evidence shows that some women report the appearance and recrudescence of depressive symptoms, evidencing the need of further studies to identify the risk factors in these cases. The importance of clear and simple information and a shared decision on which contraception to choose is crucial, as clarification about their interactions with psychiatric treatment. The clinician must also be aware of the implications for reproductive health, in order to reduce the risks and side effects associated with some drugs.

**Disclosure of Interest:**

None Declared

